# Determination of baloxavir marboxil in pharmaceutical preparations and spiked human plasma using its quenching action on acetoxymercuric fluorescein reagent: Assessment of greenness and whiteness

**DOI:** 10.1016/j.heliyon.2024.e32120

**Published:** 2024-05-29

**Authors:** Mohamed S. Nasr, Mohamed M.Y. Kaddah, Samir Morshedy, Gamal Omran, Wael Talaat

**Affiliations:** a Department of Pharmaceutical Analytical Chemistry, Faculty of Pharmacy, Damanhour University, Damanhour, 22514, Egypt; b Pharmaceutical and Fermentation Industries Development Center, City of Scientific Research and Technological Applications, New Borg El-Arab, 21934, Alexandria, Egypt

**Keywords:** Spectrofluorimetry, Baloxavir marboxil, Fluorescence quenching, Acetoxymercuric fluorescein

## Abstract

A straightforward, reliable, and cost-effective spectrofluorimetric approach has been established for the analysis of baloxavir marboxil (BXM) in raw material, tablets, as well as spiked human plasma. The approach relies on BXM's quenching impact on acetoxymercuric fluorescein (AMF) fluorescence intensity. To improve the reaction, factors such as AMF's concentration, solution's pH, diluting solvents, and reaction time were examined and optimized. Linearity, range, accuracy, precision, LOD, and LOQ were all verified in compliance with ICH criteria. The concentration range was shown to be linear between 0.2 and 2 μg/mL. The technique was effectively utilized for BXM analysis in both its tablet as well as spiked human plasma, with mean % recoveries of 101 ± 0.36 and 98.77 ± 0.65, respectively. Two assessment models (AGREE and RGB-12) were used to compare the proposed process's greenness and sustainability to four previously published chromatographic techniques. Higher green and sustainability qualities were declared by the suggested approach than by earlier ones.

## 1 Introduction

**Fig. 1:**
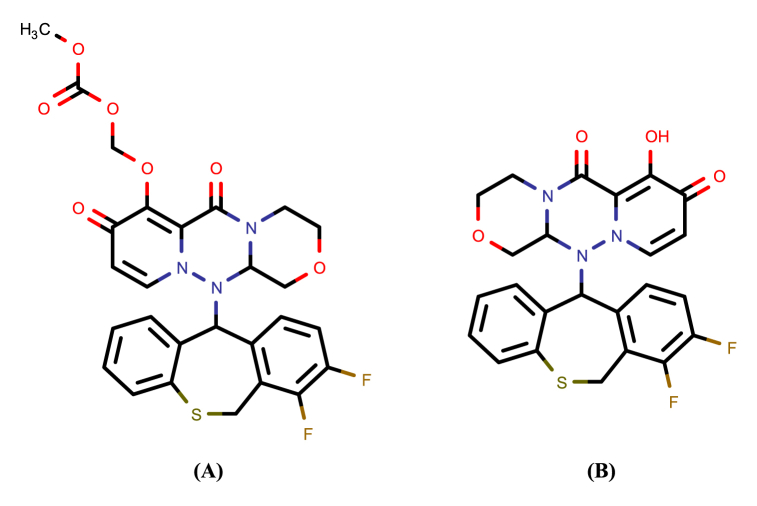
Chemical structure of (A) Baloxavir marboxil and (B) Baloxavir.

Influenza is a serious, contagious respiratory illness induced by the influenza virus. According to the WHO, influenza A and B combined can cause 3 to 5 million serious infections and up to 650,000 fatalities each year [1]. Vaccination is largely regarded as the most efficient method of preventing influenza infection [2]. Although these vaccines are available, influenza still significantly increases morbidity and death, demanding the development of antivirals to treat the disease [3]. Baloxavir marboxil (BXM) is a new influenza antiviral medication that was approved on October of 2018 for both influenza A and B treatment by the U.S Food and Drug Administration (FDA) [4]. BXM is a prodrug that, upon hydrolysis, yields the active form baloxavir. Baloxavir stops the spread of influenza viruses by blocking the endonuclease function of the polymerase acidic (PA) protein [5]. It is prescribed for individuals 12 years of age and older who have had symptoms for no longer than 48 h for the treatment of acute, uncomplicated influenza. The chemical structures of BXM and its active form, baloxavir, are displayed in Fig. 1(A, B) [6]. The solubility of BXM is 0.015 mg/mL in water and 8.6 mg/mL in methanol [6].

BXM was also tested for its efficacy against SARS-CoV-2. Recently, the goal was to incorporate BXM in therapeutic trials, either by itself or in combination with favipiravir [[7], [8], [9], [10]].

Literature review revealed that only a few chromatographic approaches have been developed for BXM analysis, including RP-HPLC [11,12], HPLC-MS [[12], [13], [14]], and LC-MS/MS [15]. As far as we know, no spectrophotometric or spectrofluorometric approaches were reported for BXM analysis. Chromatographic techniques are money and time-consuming and involve excessive usage of organic solvents as well as sophisticated instruments, which are all great limitations to these methods [16]. Fluorescence quenching technique is the reduction of a fluorophore's quantum yield of fluorescence [17]. The technique is very sensitive, quick, easy, specific, and selective, among other benefits [18,19]. Research into fluorescence quenching is convenient because of its wide-ranging applications in fields like pharmaceutical, chemical, and biological analysis. Several publications analyzing various pharmaceuticals and chemical substances using the quenching approach were published [[20], [21], [22], [23], [24], [25], [26], [27]].

**Fig. 2:**
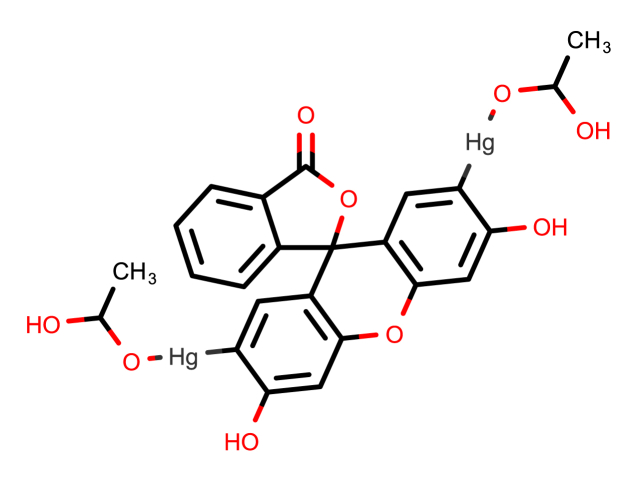
The chemical structure of AMF reagent.

In this work, we employed acetoxymecuric fluorescein (AMF), which is a widely used fluorescent reagent (Fig. 2), for the quantification of our non-fluorescent drug, BXM. The green fluorescence of AMF reagent is quenched when specific anions and groups are present, including sulfide, cyanide, iodide, and the sulfhydryl moiety [28,29]. Therefore, owing to the unique way in which these groups interact with the Hg^2+^ present in AMF's structure, AMF has been extensively employed in the quantitative identification of non-fluorescent substances containing sulfide or sulfhydryl moieties, such as penicillamine [30], timonacetic acid, acetylcysteine, and mesna [31], mirabegron [32], and rivaroxaban [33].

Our research aims to offer a spectrofluorimetric method that is accurate, quick, easy, sensitive, and affordable for the quantification of BXM in both its tablets and spiked human plasma. As far as we're aware, the current technique is the first spectrofluorimetric approach for BXM analysis.

The developed approach has several advantages over the reported ones since it is easier to use, less expensive, and does not involve intricate procedures or pricey detectors like those used in chromatographic and mass spectrometric techniques. Moreover, the greenness and sustainability of the proposed approach were evaluated and compared to those of the reported ones, and the developed approach provided satisfactory results.

## 2 Materials and methods

### 2.1 Materials and reagents

BXM pure powder was obtained from HRV Global Life Sciences, Telangana, India. Xofluza® tablets (Lot No. 3388332; manufactured by Shionogi Pharma, Tokyo, Japan) containing 20 mg BXM were bought from a local drugstore. All reagents and chemicals employed were of analytical grade. Spectrophotometric grade of methanol (99.9 %) and acetonitrile (99.5 %) were bought from Sigma Aldrich (Taufkirchen, Germany), while isopropanol, chloroform, and diethylether were bought from El-Nasr Co. (Cairo, Egypt). Our research facility provided us with distilled water using WS-100 series Stuart water stills (Cole-Parmer, Vernon Hills, USA) and was employed during all experiments. Equivalent volumes of 0.1 M of phosphoric acid, 0.1 M of acetic acid, and 0.1 M of boric acid were combined to prepare Britton Robinson buffer, which was then pH-adjusted to the necessary range (2–9) using 0.1 M sodium hydroxide. Human plasma samples were obtained from the blood bank (Medical Research Institute, Alexandria University, Alexandria, Egypt).

### 2.2 Instrumentation

All spectrofluorometric spectra were implemented using the Cary Eclipse Spectrofluorimeter (Agilent, Santa Clara, CA, USA). It employs a Xenon flash lamp (80 Hz) as a light source (200–900 nm) and is remotely controlled using Cary WinFLR software. The instrument was frequently checked for calibration and linearity using a 0.01 μg/mL standard solution of quinine sulfate at excitation and emission wavelengths of 255 and 378 nm, respectively. All pH readings were taken with a digital pH meter, Jenway 3310 (Cole-Parmer, Vernon Hills, USA). The temperature effect was studied using a digital precise shaking water bath (Bio Techno Lab, Mumbai, India).

### 2.3 Preparation of standards and stock solutions

BXM's stock solution of a concentration of 100 μg/mL was made by dissolving 10 mg of BXM in 100 mL methanol. The prepared stock solution was further diluted using distilled water in order to obtain the working solutions.

A 1 × 10^−4^ M solution of AMF reagent was obtained by dissolving 84.9 mg of the AMF powder in 20 mL of 0.1 M sodium hydroxide, diluting with 100 mL of 0.1 M solution of boric acid, and completing the volume to 1.0 L by distilled water [29,31,32]. All prepared solutions were protected from light and refrigerated.

### 2.4 General procedures 2.4.1. construction of the calibration curve

The presented technique was used under optimal conditions, which we will discuss further below. A calibration curve was obtained for BXM analysis by plotting the differences in the reagent's fluorescence intensity versus the quencher's concentration, which is BXM. The reaction mixture was prepared by transferring accurate volumes of drug working solution (10 μg/mL) into a series of 10-mL calibrated volumetric flasks in order to achieve a baloxavir concentration range of 0.2–2.0 μg/mL, then 1 mL of AMF reagent was added into each flask. All solutions were then mixed using a vortex, and the volumes were filled to mark with distilled water to achieve the required concentrations. Following excitation at *λ*_ex_ 498 nm, the reagent's fluorescence was then measured at *λ*_em_ 520 nm. After that, the difference in fluorescence was computed by deducting the emission intensities of the prepared reaction mixes from values that corresponded to the blank samples that had been similarly handled (solution containing 1 mL of AMF solution and completed to the mark using distilled water).

#### 2.4.1 Analysis of Xofluza tablets

The weight of ten Xofluza® tablets was recorded, and the average of those readings was determined. After thoroughly grinding all of the tablets, the equivalent of one tablet's weight (20 mg) was moved to a 100-mL calibrated flask then sonicated for 10 min with methanol (30 mL). After that, the flask was filled to the mark using the same solvent. The resulting solution was filtered, and distilled water was used for further dilution of the filtered solution, yielding a working solution for BXM of concentration of 10 μg/mL. The method was conducted in the same manner as described in Section 2.4.1. Using either the calibration plot or the associated regression equation, the nominal concentration was computed and obtained.

#### 2.4.2 Procedures for studying the calibration curve in spiked human plasma

Plasma samples were kept at −80 °C. Before handling, they were allowed to thaw at room temperature as instructed. Aliquots of plasma samples of 1 mL each were added to a series of centrifugal tubes and spiked with 1 mL of varied concentration working stock solutions of BXM, then vortexed for 30 s. Spiked samples were then extracted with 5.0 mL diethyl ether and centrifuged at 8000 rpm for 30 min. The organic ether layers were carefully isolated. The isolated layers were then dried by evaporating them under a stream of nitrogen gas. The obtained residues were dissolved in 5-mL distilled water to achieve the needed concentrations (0.25–2 μg/mL). The same steps under Section 2.4.1 were used that were described for constructing the calibration graph. All procedures were performed in compliance with institutional guidelines and were approved by the Ethics Committee of the Faculty of Pharmacy, Damanhour University, Egypt (Ref. No. 621PA18).

## 3 Results and discussion

**Fig. 3:**
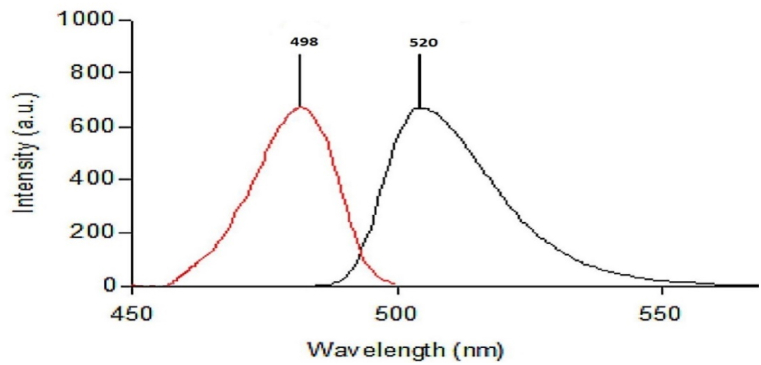
Excitation and emission spectra generated using 1 mL of AMF solution (1 × 10^−4^ M) at 498 and 520 nm, respectively.

**Scheme 1:**
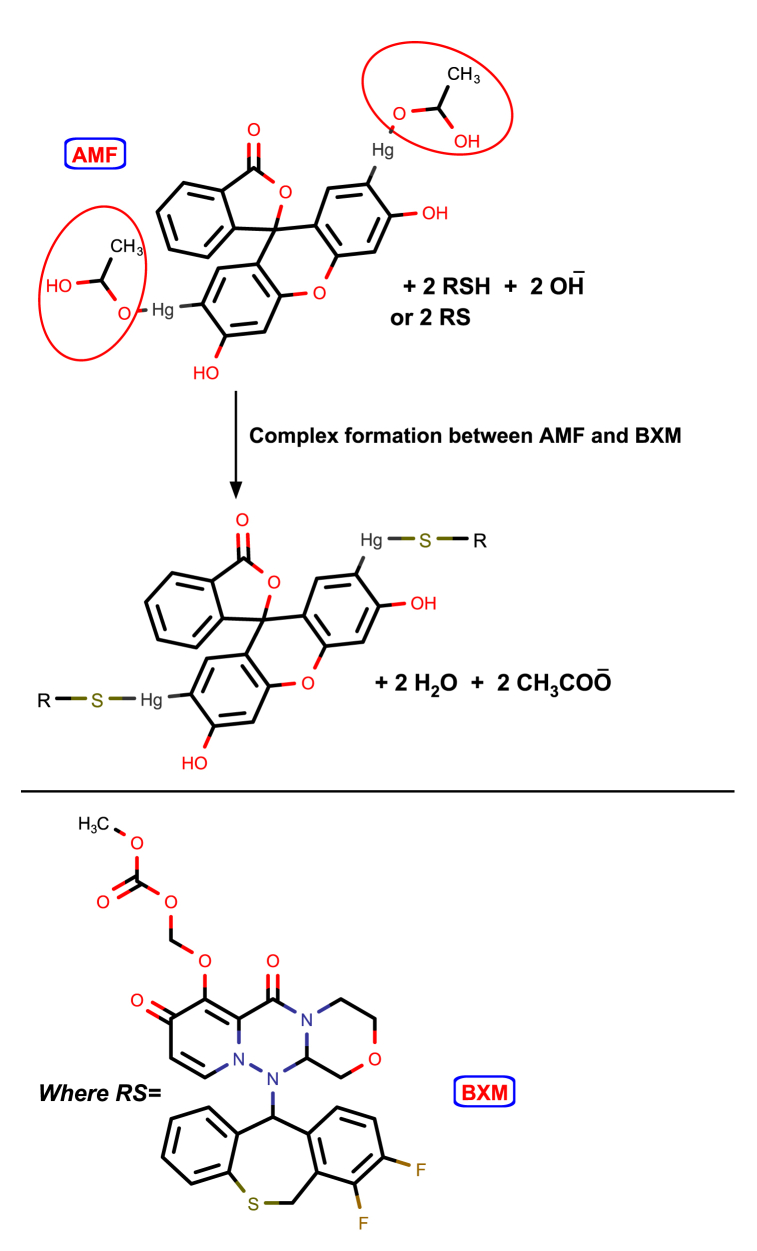
The suggested mechanism of the AMF-BXM reaction.

AMF's prepared solution is yellow in color and emits green-colored fluorescence at an emission wavelength of 520 when activated in an alkaline medium at an excitation wavelength of 498 nm. The excitation and emission spectra of 1 mL of 1 × 10^−4^ M AMF reagent's solution are displayed in Fig. 3. Our work is based on the ability of BXM to quench the fluorescence of AMF reagent. Though the exact mechanism of the reaction between mercury in the AMF structure and thiol compounds is unknown, complex formation is thought to be a likely pathway [29,32,34]. This is known as the Wronski reaction [28]. Hubicki et al. proposed that sulfur and mercury form a coordination linkage [35], while Simon et al. postulated that mercury forms thiolate bridges with sulfur due to the strong Hg - S atom pair analysis [34]. According to the suggested reaction mechanism [[29], [30], [31], [32], [33]], which is depicted in Scheme 1, this would result in the formation of a stable complex between BXM and AMF reagent, changing the reagent's chromophore and thereby reducing the intensity of its fluorescence.

### 3.1 Optimizing reaction's conditions

To achieve the highest degree of sensitivity, many reaction-affecting factors, such as AMF reagent's concentration, the ideal pH, the reaction's time, and the ideal diluting solvent, were studied and tuned.

#### 3.1.1 AMF's concentration effect

Various volumes (0.2–2 mL) of a 1 × 10^−4^ M of AMF solution were used to interact with a fixed drug concentration in a series of 10 mL calibrated flasks in order to examine the influence of AMF solution concentration. For each flask, the fluorescence difference between each mixture solution and blank solution was recorded at 520 nm as λem after excitation at 498 nm. Fig. S1 demonstrates the impact of AMF's volume on the mixture's fluorescence difference. The difference in fluorescence increased progressively until it reached 1 mL of AMF and then remained steady until it reached 1.6 mL. After that, as AMF's volume increased, the difference in fluorescence declined. This observed decline in fluorescence is credited to the inner filter effect, in which high concentration changes the incident light intensity that reaches each molecule [36]. Therefore, 1 mL of 1 × 10^−4^ M AMF was selected as the optimum reagent volume.

#### 3.1.2 Effect of solution's pH

The pH is a significant key parameter influencing the sensitivity of the reaction. Using Britton Robinson buffer, the influence of pH on the drug's ability to quench AMF's emitted fluorescence was investigated over the pH range of 5–9 (Fig. S2). Additionally, the initial reaction mixture's pH was determined to be 6.8. It can be clearly observed that AMF's maximum fluorescence intensity occurred between 6.5 and 7 pH values, owing to AMF's existence in the form of a strongly fluorescing, doubly charged anion [29,31,33]. When the pH rose above 7.0 or fell below 6.0, non-fluorescent forms of AMF started to develop, causing the AMF fluorescence to diminish. For feasibility, the reaction was conducted at the original pH of the solution (6.8)

#### 3.1.3 Effect of time on quenching

The impact that the reaction time had on BXM's quenching effect was investigated in this study (Fig. S3). The quenching was monitored every 5 min for 30 min. It was observed that BXM reacts instantly with AMF and that the greatest quenching response is attained on direct mixture measurement after preparation; thus, the mixture was measured immediately after preparation.

#### 3.1.4 Effect of diluting solvent

The impact of several diluting solvents with different polarities, including distilled water, chloroform, isopropanol, methanol, and acetonitrile, on the quenching process was investigated (Fig. S4). Distilled water was found to be the best solvent as it gave maximum sensitivity. This may be explained by the fact that higher solvent polarity reduces the π-π* energy transition but increases the n- π* energy transition because polarity induces the stabilization of the excited state, which has a higher momentum than the ground state. The increased excited state stability enhances the excited state electron populations, which in turn raises the fluorescence intensity [37]. As a result of being the most polar of the solvents examined, distilled water offered the maximum sensitivity of all those studied.

**Fig. 4:**
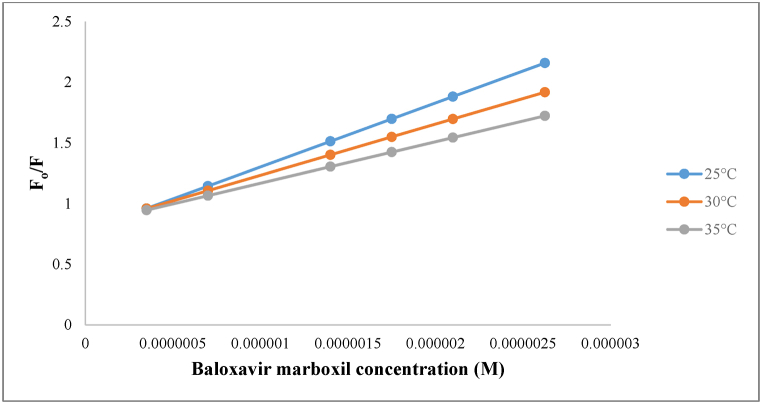
Stern-Volmer plot for AMF-BXM interaction at various temperatures (25, 30, and 35 °C).

Following the completion of the reaction's conditions optimization, the developed quenching interaction mechanism was inspected using the Stern–Volmer plot. This was done by producing a curve (Fig. 4) of F_o_/F versus BXM's molar concentration (the quencher). Where F_o_ and F are AMF's fluorescence intensity without and with BXM (quencher), respectively. The obtained Stern–Volmer plot demonstrated a linear relationship, indicating that the quenching mechanism for the reagent-drug interaction is either static or dynamic. To determine whether the mechanism is static or dynamic, a temperature study was performed by plotting the Stern–Volmer plot at various temperatures (25, 30, and 35 °C) (Fig. 4). It was observed that the plot's slope decreased, which in turn indicates that the quenching decreased as the temperature increased, proving that the quenching process between AMF and BXM takes place through a static mechanism, confirming complex formation between the drug and the reagent. The association constant (Ka) was computed according to the following equation (Eq. (1)) [38], and it was found to be 5.27 × 10^5^.

(1) F °/ F = 1 + K_a_[Q]

‘F°’ is AMF's fluorescence intensity in BXM's (quencher) absence, whereas ‘F’ is its fluorescence intensity in BXM's presence. Ka is the association constant, while [Q] is the BXM's molar concentration [38].

**Fig. 5:**
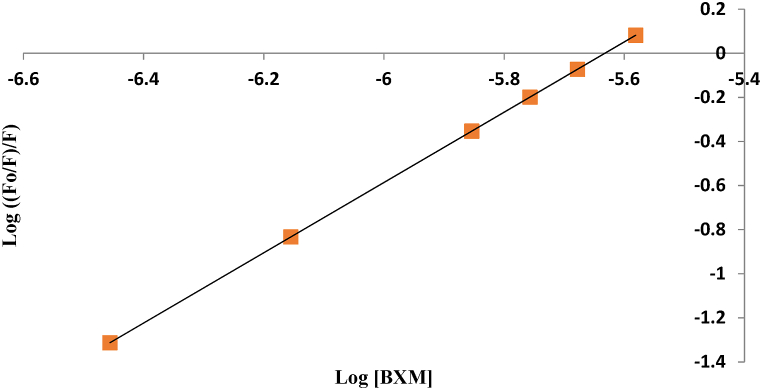
Modified Stern-Volmer plot for BXM interaction with the AMF.

The number of BXM's binding sites with the reagent was calculated using the modified Stern–Volmer plot's slope (Fig. 5) [23]. The slope was found to be 1.59 (Fig. 5), which shows that BXM and AMF have two binding sites, indicating a ratio of 2: 1 (BXM: AMF).

##### 3.1.4.1 UV–Visible absorption spectra study

**Fig. 6:**
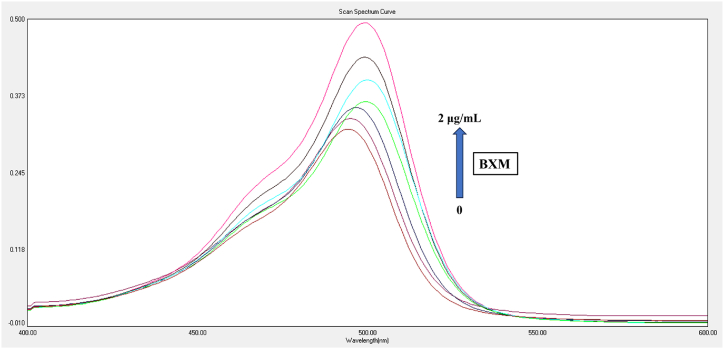
UV spectra of AMF reagent with different concentrations of BXM.

Utilizing UV–vis absorption to investigate interactions between reagents and drugs, as well as the formation of ground state complexes, is a straightforward and easily affordable technique [[39], [40], [41], [42], [43], [44]]. UV–vis spectra can be used to distinguish between dynamic and static quenching. More specifically, the fluorophore's absorption spectra are unaltered during dynamic quenching while it is continuously changing during static quenching. The UV–vis absorption spectrum of AMF reagent was analyzed with varying concentrations of BXM (Fig. 6). The results showed a shift in the absorption maximum at 498 nm and an increase in peak intensity (hyperchromic shift) as the BXM concentration increased. This indicates that the fluorescence quenching of AMF by BXM involves static quenching through the formation of a drug-reagent complex.”

### 3.2 Validation of the proposed method

According to ICH requirements, the new technique was verified regarding range, linearity, accuracy, precision, LOD, and LOQ [45]. Table 1 lists all of the validation parameters.

**Table 1:** Regression parameters for the analysis of BXM by the suggested spectrofluorometric procedure in raw material and spiked human plasma.

Parameters	Raw material	Spiked human plasma
**λ**_**ex**_**& λ**_**em**_**(nm)**	498 & 520	498 & 520
**Linearity range (μg/mL)**	(0.20–2.00)	(0.25–2.00)
**LOD (μg/mL)**	0.026	0.033
**LOQ (μg/mL)**	0.085	0.110
**Slope ± S**_**b**_	267.49 ± 1.85	273.91 ± 2.43
**Intercept ± S**_**a**_	−17.81 ± 2.31	−8.78 ± 3.06
**%RSD**	0.69	0.89
**Regression equation**	*ΔY 520* = *267.49C - 17.81*	*ΔY 520* = *273.91C - 8.78*
**Significance *F***	7.34 × 10^−12^	3.33 × 10^−11^
**Correlation coefficient (*r)***	0.9997	0.9996

#### 3.2.1 Range and linearity

The new method's linearity was tested by assessing varying concentrations of a series of solutions under previously adjusted conditions. The resulting fluorescence quenching response proved to be linear related to BXM's concentration over the range (0.2–2 μg/mL) for the bulk powder as well as (0.25–2 μg/mL) for spiked human plasma. Figs. S5 and S6 present the calibration curves that correlate the difference in fluorescence between the signals from AMF reagent blank solutions and the ones obtained upon quenching by BXM to the respective drug's concentrations in μg/mL in bulk powder and spiked human plasma, respectively. The correlation coefficients (*r*), the intercepts, the slopes, the standard deviations of the intercept, and the standard deviations of the slope were all determined as part of the regression analysis that was implemented using the least squares method on the calibration data (Table 1). The findings validated the suggested method's satisfactory linearity, as evidenced by the high *r* value (≥0.9996), % RSD of the slope (<2 %), and low significance *F* value, indicating the low dispersion degree of experimental point around the fitted regression line. The plotted points' closeness to the straight line is demonstrated by the residuals' standard deviation (S_y/x_) low value, which was also calculated.

#### 3.2.2 Limit of detection (LOD) and limit of quantitation (LOQ)

LOD and LOQ are set at 3.3S/b and 10S/b, respectively, where S represents the standard deviation of intercept while b is the calibration line's slope. The low LOD and LOQ values (Table 1) proved the suggested approach's great sensitivity for BXM analysis.

#### 3.2.3 Accuracy and precision

In accordance with ICH guidelines, we assessed accuracy as well as intra- and inter-day precisions to assure the reliability and repeatability of the suggested method. Three measurements per each concentration level within the range of linearity were taken (Table 2). % Recovery as well as % RSD were calculated for each level. Accuracy and intraday precision were determined on a single day, while inter-day precision was evaluated by carrying out the same concentration level three times on three separate days. The acquired results fell within the accepted ranges of 98–102 % for recoveries and ±2 % for % RSD, confirming the method's accuracy and precision.

**Table 2:** Intra-day and inter-day accuracy and precision for the analysis of BXM using the proposed method.

Intra-day				Inter-day		
Conc. (μg/mL)	Found ± SD	Accuracy (%R)	Precision (%RSD)	Found ± SD	Accuracy (%R)	Precision (%RSD)
0.20	0.199 ± 0.003	99.83	1.76	0.197 ± 0.003	98.33	1.55
1.00	1.009 ± 0.018	100.93	1.78	1.016 ± 0.016	101.60	1.61
2.00	2.023 ± 0.026	101.13	1.29	2.029 ± 0.037	101.47	1.82

#### 3.2.4 Robustness

The suggested approach's robustness was confirmed by studying the effect of deliberate small deviations in various conditions, including *λ*_ex_ (498 ± 2 nm), *λ*_em_ (520 ± 2 nm), volume of AMF solution (±0.1 mL) and pH (±0.2 units). Small RSD% values (<2 %) support that these deviations did not have any notable influence on the analysis of BXM using the proposed method (Table S1).

#### 3.2.5 Selectivity, specificity, and matrix effect

The proposed method's selectivity was evaluated through the analysis of the co-administered medications that are commonly used during influenza infection, including antivirals such as oseltamivir and analgesics and antipyretics such as aspirin, paracetamol, and ibuprofen. All tested drugs caused no quenching of the fluorescence of the reagent as they lack a sulfide moiety in their structures and therefore don't interfere with BXM determination. Excipients that are commonly used, like lactose, mannitol, carboxymethyl cellulose, cellulose, and sucrose, were spiked with BXM, and the AMF's fluorescence intensity was measured. AMF's fluorescence intensity was not quenched by the previously specified excipients, which is a sign of good technique specificity. By inspecting the plasma extract's fluorescence spectra, the matrix effect's interference was evaluated. As the blank matrix does not affect the reagent's fluorescence spectrum, the drug's extraction from plasma was demonstrated to be selective.

#### 3.2.6 BXM and AMF stock solutions stability

BXM solution (1 μg/mL) and AMF solution (1 × 10 ^−4^ M) were made in two sets, one of which was stored in a refrigerator and the other at room temperature. For 12 h, the solutions were examined hourly. Then, for 14 days, they were examined every 24 h. The solutions were shown to be stable for 8 days at room temperature and 15 days in the refrigerator, according to the obtained results.

#### 3.2.7 Final solutions ready for response measurement stability

The final solutions’ stability was inspected for 2 h at room temperature. On immediate measurement, the fluorescence difference was determined to be at its peak and stayed steady for 1 h.

### 3.3 Analytical applications

#### 3.3.1 The analysis of BXM raw materials and its pharmaceutical preparation

The suggested technique was successfully employed for BXM determination in raw materials as well as in its commercial dosage form, Xofluza® tablets. Methanol was used for the extraction of the active ingredient and diluted with distilled water to achieve concentration levels within the indicated ranges. The assay results presented in Table 3 showed high accuracy and precision and excellent agreement with the label's claim, as demons**t**rated by % recovery, % RSD, and % Er values. The results for BXM determination in Xofluza® tablets exhibited a good agreement with the findings acquired from the HPLC approach in comparison [13], as indicated by the values of the student's *t*-test and variance ratio *F*-test presented in Table 3. Since the estimated values did not surpass the theoretical ones, there was no appreciable dissimilarity in accuracy and precision between the developed approach and the comparison one.

**Table 3:** Assay results for the analysis of BXM in Xofluza tablets using the proposed and reported method.

Proposed Method		Reference Method [13]	
Concentration (μg/mL)	% Found	Concentration (μg/mL)	% Found
0.20	101.41	8.00	100.22
1.00	100.42	30.00	100.70
2.00	100.92	60.00	101.11
mean	100.91		100.68
SD	0.50		0.45
***F-test***	**1.21**	**(19)**^a^	
***t-test***	**0.63**	**(2.78)**^a^	

a The value of tabulated *t* and *F*, (at *p* = 0.05).

#### 3.3.2 The analysis of BXM in spiked human plasma

The new spectrofluorimetric method's high sensitivity made it possible to analyze BXM in spiked human plasma. To overcome matrix interference, the samples were exposed to a liquid-liquid extraction (LLE) clean-up process. In this respect, the LLE of BXM was executed using diethyl ether. A calibration curve of spiked human plasma was constructed (Table 1) for further BXM in-vivo analysis in real human plasma. This approach was applied effectively to analyze BXM in spiked plasma, with a mean % recovery of 98.77 ± 0.65. Table 4 presents the findings of the analyses conducted on spiked plasma.

**Table 4:** Analysis of BXM in spiked human plasma by the proposed approach.

Spiked amount in (μg/mL)	% Recovery
0.25	98.15
1.00	98.70
2.00	99.45
**Mean** ± **SD**	**98.77 ± 0.65**

### 3.4 Greenness and whiteness assessment of the proposed techniques

It has become increasingly important for the scientific community to incorporate green analytical chemistry (GAC) as well as white analytical chemistry (WAC) principles into their everyday work so that they can achieve an appropriate balance between the method's greenness, its usefulness, and the practical and financial considerations [[46], [47], [48], [49], [50], [51]]. Multiple indicators have been created in recent years to assess the environmental impact of analytical techniques. In order to compare and rank various analytical methods in terms of their greenness, performance, applicability and cost more accurately. In our investigation, the innovative Analytical Greenness Metric (AGREE) [52] was employed to judge the developed approach's greenness. We tried strategically to prevent factors that could potentially result in lower scores. For example, we avoid employing heating during technique development and also choose to fill flasks with distilled water rather than organic toxic solvents. Moreover, fluorometric devices are characterized by their lower energy consumption compared to chromatographic techniques. These conscious decisions contribute to greater ratings as compared to traditional approaches, coinciding with sustainability goals. WAC is an emerging field that aims to complement green analytical chemistry by taking a more comprehensive approach. WAC is more in line with the idea of sustainable development since it seeks a compromise that prevents increasing greenness unconditionally at the cost of functionality [53,54]. For this purpose, the RGB-12 tool [53] was used as a multi-criteria assessment method for the evaluation of the whiteness of the proposed approach.

The proposed spectroflouremetric method's greenness and whiteness profiles were carefully compared and graded with those of the previously published HPLC-PDA [11], UHPLC-PDA [14], HPLC-MS [13], and LC-MS/MS [15].

#### 3.4.1 The AGREE tool

AGREE is currently the most used tool for assessing greenness [52]. It operates using a calculator, which may be downloaded from (https://mostwiedzy.pl/AGREE.). The AGREE calculator incorporates the 12 principles of green analytical chemistry (GAC). The ultimate score, which ranges from 0 to 1, is represented by the AGREE pictogram's center. The central result-associated color represents the color combination showing the 12 AGREE pictogram sectors' performance. The optimal approach with the highest score, 1, is shown as a dark green color. Consequently, the AGREE tool is considered simple, convenient, and extremely fast. The AGREE pictograms presented in Table 5 compare the performance of the developed spectrofluorimetric approach with the other four reported approaches. Where the AGREE scores for the five evaluated methods were found to be 0.60 for the developed spectrofluorimetric approach, 0.59 for the HPLC-PDA [11] and UPLC-PDA approaches [14], 0.56 for the HPLC-MS approach [13], and 0.54 for the LC-MS/MS approach [15].

**Table 5:**
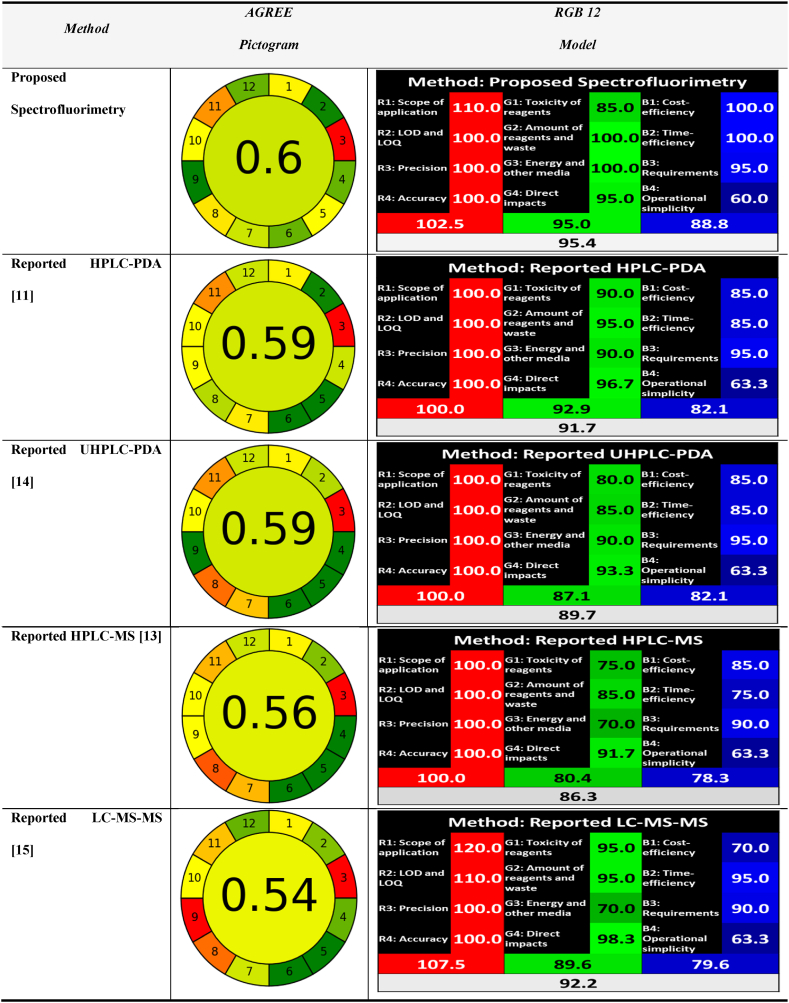
Greenness assessment and comparison of the developed method and reported methods using AGREE and RGB-12 metrics.

A thorough analysis of the five analytical processes AGREE scores shows both positive and negative green characteristics in different sections:

*Section 1 (Sample Handling)*: Due to offline analysis steps, all five methods received a yellow-colored rating.

*Section 2 (Minimal Sample Size)*: All five methods receive different shades of green rating due to their minimal sample requirement.

*Section 3 (In Situ Measurements)*: All five approaches receive red ratings since they involve offline analytical procedures.

*Section 4 (Sample Preparation Steps)*: The five approaches show varying shades of green ratings depending on how many steps each of their different processes requires.

*Section 5 (Automation)*: All four reported chromatographic methods receive a green rating due to the automatic nature of their devices as they employ an autosampler. On the other hand, our developed spectrofluorimetric approach receives a yellow rating for being manual in nature.

*Section 6 (Derivative Reagents)*: All four reported chromatographic methods receive dark green ratings as they don't utilize any derivatizing agents. On the other hand, our developed spectrofluorimetric approach receives a light green rating as it utilizes one derivatizing agent, AMF (CAS number: 3570-80-7).

*Section 7 (Analytical Waste Volume)*: The developed spectrofluorimetric and the reported LC-MS/MS approaches [15] receive light green ratings as they produce minimum amount of waste. On the other hand, the other three reported methods receive yellow to orange ratings as they have longer run times and consume larger amounts of solvents leading to the production of larger amounts of wastes.

*Section 8 (Multi-Analyte Methods)*: This section evaluates two different criteria; the first one is the number of analytes analyzed per run, while the second one evaluates the number of samples analyzed per hour. All approaches determine only one analyte except the HPLC-PDA approach [11], which quantify BXM in addition to five of its impurities. Additionally, all approaches have comparable preparation steps duration, with the chromatographic methods having a longer run time compared to the spectrofluorimetric method. Consequently, the HPLC-PDA approach receives a light green rating as it shows the highest number of analytes analyzed per run, followed by the developed spectrofluorimetric method, which receives a yellow rating, and then the three chromatographic methods, which receive an orange rating as they have fewer samples analyzed per hour.

*Section 9 (Energy Consumption)*: The developed spectrofluorimetric method scores green due to the remarkably lower energy consumption of the spectrofluorometric instruments compared to the chromatographic instruments, especially those coupled with mass spectrometer, which consumes a much higher amount of energy and consequently receives red ratings.

*Section 10 (Reagents from Renewable Sources)*: All five approaches receive yellow ratings since they employ some bio-based reagents.

*Section 11 (Hazardous Solvents)*: All five approaches receive orange ratings for their choices of solvents.

*Section 12 (Operator Safety)*: All five approaches receive varying shades of green ratings regarding operator safety.

In conclusion, the developed spectrofluorimetric method had a satisfactory green AGREE score among the five approaches, owing to the usage of distilled water, its lower energy consumption, as well as lower waste generation.

The AGREE program highlights areas for environmental friendliness enhancement while providing a thorough understanding of analytical techniques. It does not, however, take into account the importance of accuracy, precision, and repeatability in achieving a balance between environmental effect and analytical performance, which calls for the adoption of an additional assessment tool. In this regard, the RGB-12 tool was applied.

#### 3.4.2 Red green blue (RGB-12) model

**Fig. 7:**
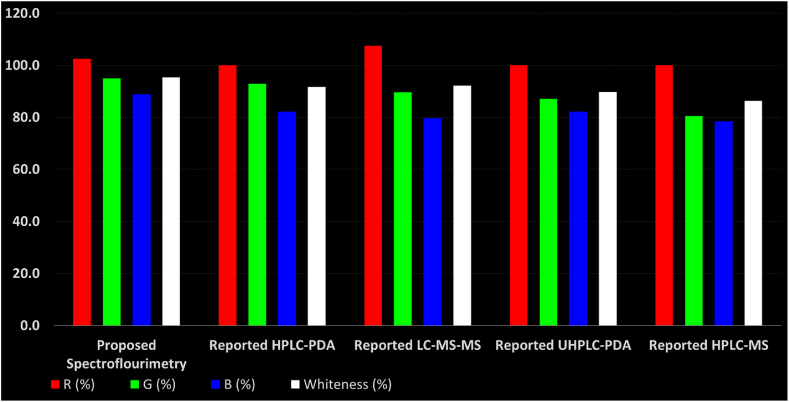
The main evaluation outcomes resulted from the RGB12 comparative study for the proposed method together with the published methods. The white bar indicates the arithmetic mean of the three other bars (red, green, and blue). The scores above 100 % indicate extra performance exceeding the basic requirements.

The RGB 12 model is divided into three distinct groups [53]. Each group is denoted by a different color, and inside it are a set of parameters that, when combined, evaluate significant features of the analytical method. The red region assesses analytical performance based on validation standards, including precision, accuracy, LOD and LOQ, and application scope. The recognized GAC concepts are assigned to the green region, while productivity factors including time and cost effectiveness, minimum practical demands, and operational simplicity are represented in the blue region. The RGB-12 model is displayed as an Excel sheet that adheres to the white analytical chemistry (WAC) values. The methodologies are plainly evaluated in line with the 12 WAC principles, and the degree of sustainability as determined by the whiteness assessment is estimated. An analytical approach that is both thorough and practical is considered "white" by proponents of the WAC methodology. The developed approach was investigated and objectively compared to the other four reported procedures. Table S2 and Fig. 7 summarize the outcomes of all of these approaches' evaluations employing the innovative RGB-12 model.

For the validation criteria (red zone), all five methods seemed to be appropriate for their target application, and therefore they graded 100 in all aspects of the analytical performance (red) zone [53]. Regarding the scope of application, the proposed spectrofluorimetric method received extra points (110) since it was applied for BXM analysis in spiked human plasma and can be extended for in vivo drug analysis, while the reported LC-MS/MS [15] received the highest score (120) since its scope of application was extended to be applied for in vivo pharmacokinetic study. Regarding the LOD and LOQ aspect, the reported LC-MS/MS received extra points (110) as it has the lowest LOD value. All techniques were found to be accurate and precise.

For the environmental characteristics (green zone), the suggested spectrofluorimetric method was shown to be the most environmentally friendly as it had the highest score (95 %) with the least waste production and energy usage, followed by the reported HPLC-PDA (92.9 %) [11], LC-MS/MS (89.6 %) [15], UHPLC-PDA (87.1 %) [14], and HPLC-MS (80.4 %) [13], which was found to be the least environmentally friendly. Both animals and genetically modified organisms (GMOs) were not used in any of the study protocols.

For the sustainability and productivity (blue zone), the developed spectrofluorimetric method had the highest score (88.8 %) since it was the most cost-effective, time-efficient, and simple to use. Both the reported HPLC-PDA [11] and UHPLC-PDA [14] methods came in second with a lower score (82.1 %) as they were more sophisticated to manipulate and required more time than the developed method. The reported LC-MS/MS [15] and HPLC-MS [13] methods came in third (79.6 %) and fourth (78.3 %), respectively, which can be attributed to the larger amount of solvents and reagents used during analysis, the need for skilled operators and the longer time required for analysis.

The findings of this thorough investigation on whiteness are displayed in Table 5 and S2, as well as Fig. 7. The combined results demonstrated that the proposed spectrofluorimetric method is top white with overall scores of 95.4 %. The reported LC-MS/MS [15] method came in second with a lower overall score of 91.7 % as it had the highest red regions score (107.5 %) while being the lowest in terms of greenness (89.6 %) and productivity (79.6 %), then the reported HPLC-PDA [11] and UHPLC-PDA [14] methods came in third and fourth, with overall scores of 91.7 % and 89.7 %, respectively. Finally, the reported HPLC-MS [13] method came in last with an acceptable overall whiteness score of 86.3 %.

## 4 Conclusion

AMF reagent is an excellent fluorophore with an emission fluorescence peak at 520 nm following excitation at 498 nm. The proposed method depends on BXM's ability to quench AMF's emitted fluorescence. The reagent was employed for the quantitation of BXM in Xofluza ® tablets and in spiked plasma. The proposed approach's linearity, range, accuracy, precision, LOD, LOQ, and robustness were all statistically validated. All studied parameters were confirmed to be within the accepted limits. Acceptable recoveries and a correlation coefficient (r) of ≥0.9997 indicate that the linearity and range are extremely specific. In addition, the new approach is very sensitive and appropriate for accurate measurement of low drug concentrations (0.2–2 μg/mL). The proposed method was evaluated in terms of greenness and whiteness using the AGREE and RGB-12 tools and compared to several reported methods and showed satisfactory results. Moreover, the proposed technique can be extended for BXM in vivo plasma analysis.

## Data availability

No data associated with this study has been deposited into a publicly available repository. Data included in article/supp. material/referenced in article.

## CRediT authorship contribution statement

**Mohamed S. Nasr:** Writing – original draft, Software, Formal analysis, Data curation. **Mohamed M.Y. Kaddah:** Writing – review & editing, Supervision. **Samir Morshedy:** Writing – review & editing, Supervision. **Gamal Omran:** Supervision. **Wael Talaat:** Writing – review & editing, Validation, Supervision, Conceptualization.

## Declaration of competing interest

The authors declare that they have no known competing financial interests or personal relationships that could have appeared to influence the work reported in this paper.
