# Supplementary material for: Determination of baloxavir marboxil in pharmaceutical preparations and spiked human plasma using its quenching action on acetoxymercuric fluorescein reagent: Assessment of greenness and whiteness
Source: Heliyon. 2024 May 29;10(11):e32120. doi: 10.1016/j.heliyon.2024.e32120 (PMC11190548; doi:10.1016/j.heliyon.2024.e32120)
Supplement: Multimedia component 1 [file mmc1.docx]

**Determination of Baloxavir Marboxil in Pharmaceutical Preparations and Spiked Human Plasma Using its Quenching Action on Acetoxymercuric Fluorescein Reagent: Assessment of Greenness and Whiteness**

Mohamed S. Nasr ^1,*^, Mohamed M. Y. Kaddah ^2^, Samir Morshedy ^1^, Gamal Omran^1^, Wael Talaat ^1^ *^1^Department of Pharmaceutical Analytical Chemistry, Faculty of Pharmacy, Damanhour University, Damanhour 22514, Egypt
^2^Pharmaceutical and Fermentation Industries Development Center, City of Scientific Research and Technological Applications, New Borg El-Arab 21934, Alexandria, Egypt*

*Corresponding author:*

*Mohamed S. Nasr*

*Department of Pharmaceutical Analytical Chemistry,*

*Faculty of Pharmacy, Damanhour University,*

*Damanhour 22514, Egypt*

*E-mail:* [mohamed.nasr@pharm.dmu.edu.eg](mailto:mohamed.nasr@pharm.dmu.edu.eg)

*Mob: 002 01006314419*

**Fig S1.** Effect of AMF volume on the fluorescence difference through reaction with 1 µg/mL baloxavir marboxil at 520 nm.

**Fig S2**. Effect of pH on the fluorescence quenching of 1mL AMF through the reaction with 1 µg/mL baloxavir marboxil at 520 nm.

**Fig S3.** Effect of time on the fluorescence quenching of 1mL AMF through reaction with 1 µg/mL baloxavir marboxil at 520 nm.

**Fig S4.** Effect of solvent type on the fluorescence difference of 1mL AMF through reaction with 1 µg/mL baloxavir marboxil at 520 nm.

**Fig. S5.** Calibration graph of 0.2-2.0 μg/mL using standard BXM with 1 mL of 1×10^-4^ M AMF solution at λem 520 nm.

**Fig. S6.** Calibration graph of 0.25-2.0 μg/mL BXM spiked in plasma with 1 mL of 1×10^-4^ M AMF solution at λ_em_ 520 nm.

**Table S1:** Robustness of the proposed spectroflourimetric method

| **Parameters** | **%RSD** |
| --- | --- |
| λex (498 ± 2 nm) | 1.14 |
| λem (520 ± 2 nm) | 1.25 |
| AMF volume (± 5 μL) | 1.31 |
| pH (± 0.2 units) | 1.16 |

**Table S2:** Comparison of whiteness evaluation of the introduced spectrofluorometric approach along with the four reported methods using RGB-12 model

| ***Method number*** | ***Method name*** | ***R (%)*** | ***G (%)*** | ***B (%)*** | ***Whiteness (%)*** |
| --- | --- | --- | --- | --- | --- |
| **1** | **Proposed Spectrofluorometric** | 102.5 | 95.0 | 88.8 | **95.4** |
| **2** | **Reported LC-MS-MS [15]** | 107.5 | 89.6 | 79.6 | **92.2** |
| **3** | **Reported HPLC-PDA [11]** | 100.0 | 92.9 | 82.1 | **91.7** |
| **4** | **Reported UHPLC-PDA [14]** | 100.0 | 87.1 | 82.1 | **89.7** |
| **5** | **Reported HPLC-MS [13]** | 100.0 | 80.4 | 78.3 | **86.3** |
